# NIR-Emitting Alloyed CdTeSe QDs and Organic Dye Assemblies: A Nontoxic, Stable, and Efficient FRET System

**DOI:** 10.3390/nano8040231

**Published:** 2018-04-11

**Authors:** Doris E. Ramírez-Herrera, Eustolia Rodríguez-Velázquez, Manuel Alatorre-Meda, Francisco Paraguay-Delgado, Antonio Tirado-Guízar, Pablo Taboada, Georgina Pina-Luis

**Affiliations:** 1 Centro de Graduados e Investigación, Instituto Tecnológico de Tijuana, A.P. 1166, 22500 Tijuana, BC, Mexico; doris_e_777@hotmail.com (D.E.R.-H.); antonio.tirado@tectijuana.edu.mx (A.T.-G.); 2 Facultad de Odontología, Universidad Autónoma de Baja California, Calzada Universidad 14418, Parque Industrial Internacional, 22390 Tijuana, BC, Mexico; eustolia.rodriguez@uabc.edu.mx; 3 Instituto de Ortopedia y Banco de Tejidos Musculoesqueléticos, Universidad de Santiago de Compostela, Campus Sur S/N, E-15782 Santiago de Compostela, Spain; 4 CONACyT—Instituto Tecnológico de Tijuana, Centro de Graduados e Investigación en Química, Blvd. Alberto Limón Padilla S/N, 22510 Tijuana, BC, Mexico; malatorreme@conacyt.mx; 5 Centro de Investigación en Materiales Avanzados S. C., Departamento de Física de Materiales, Av. Miguel de Cervantes 120, Complejo Industrial Chihuahua, CP 31109 Chihuahua, Chih., Mexico; francisco.paraguay@cimav.edu.mx; 6 Grupo de Física de Coloides y Polímeros, Departamento de Física de Materia Condensada, Facultad de Física, Universidad de Santiago de Compostela, Campus Sur S/N, E-15782 Santiago de Compostela, Spain; pablo.taboada@usc.es

**Keywords:** energy transfer, alloyed quantum dots, quantum dots-dye assemblies, cytocompatibility

## Abstract

In the present work, we synthesize Near Infrared (NIR)-emitting alloyed mercaptopropionic acid (MPA)-capped CdTeSe quantum dots (QDs) in a single-step one-hour process, without the use of an inert atmosphere or any pyrophoric ligands. The quantum dots are water soluble, non-toxic, and highly photostable and have high quantum yields (QYs) up to 84%. The alloyed MPA-capped CdTeSe QDs exhibit a red-shifted emission, whose color can be tuned between visible and NIR regions (608–750 nm) by controlling the Te:Se molar ratio in the precursor mixtures and/or changing the time reaction. The MPA-capped QDs were characterized by UV-visible absorption spectroscopy, fluorescence spectroscopy, transmission electron microscopy (TEM), energy dispersive X-ray spectroscopy (EDS), and zeta potential measurements. Photostability studies were performed by irradiating the QDs with a high-power xenon lamp. The ternary MPA-CdTeSe QDs showed greater photostability than the corresponding binary MPA-CdTe QDs. We report the Förster resonance energy transfer (FRET) from the MPA-capped CdTeSe QDs as energy donors and Cyanine5 NHS-ester (Cy5) dye as an energy acceptor with efficiency (*E*) up to 95%. The distance between the QDs and dye (*r*), the Förster distance (*R*_0_), and the binding constant (*K*) are reported. Additionally, cytocompatibility and cell internalization experiments conducted on human cancer cells (HeLa) cells revealed that alloyed MPA-capped CdTeSe QDs are more cytocompatible than MPA-capped CdTe QDs and are capable of ordering homogeneously all over the cytoplasm, which allows their use as potential safe, green donors for biological FRET applications.

## 1. Introduction

Quantum dots (QDs) are considered an excellent alternative to organic fluorophores due to their unique optical properties, such as broad excitation spectra, high photostability, size-tunable emission, narrow and symmetric emission spectra, and high quantum yields (QYs) [1]. These characteristics demonstrate them as attractive materials for biomedical and technological applications, such as clinical diagnosis and labeling [2,3,4], as well as for solar energy conversion in photovoltaic devices [5,6]. 

Regarding their tentative use in the biomedical field, QDs are well-known to respond in the red to near-infrared (NIR) window (600–900 nm), which is highly advantageous for in vitro and in vivo imaging and detection [7]. NIR light easily penetrates animal tissue due to the minimum absorbance of NIR photons [8]. Ternary alloyed QDs have recently emerged as a new class of NIR emitters attracting considerable attention, due to the capability of tuning their optical emission without changing the particle size. Examples of ternary NIR QDs are CuInS_2_, AgInSe_2_, and CdTeSe [9]. Several groups have investigated the synthesis of NIR-emitting CdTeSe QDs [10,11,12,13,14]. Most of these studies are based on core-shell QD synthesis, since this type of QD shows greater photostability and QYs, but their synthesis is complicated and time-consuming. Because of this, a systematic study to obtain only core-doped QDs with high QYs and photostability is required. 

Synthetic methods involving organic solvents such as trioctylphosphine (TOP) and trioctylphosphine oxide (TOPO) [15] have produced QDs soluble in non-polar solvents exclusively. This condition limits their application in the biomedical arena. Surface modification represents an alternative synthetic strategy to obtain water-soluble and less toxic QDs [16,17]; however, it is known that ligand surface modification fails to maintain the high QYs of QDs. An important strategy is the direct synthesis of water-soluble QDs, in a single step, eliminating the superficial exchange of ligands that leads to significant decreases of the QY. This strategy has been used in the hydrothermal synthesis of water-soluble binary [18,19] and ternary [20] QDs with good results, using mercapto acids as stabilizers. In particular, Ma et al. [18,19] prepared high-quality water-soluble CdTe QDs and investigated the influence of different mercapto acids on the growth rate, size distribution, fluorescence, and stability of the QDs. Liu et al. [20] presented ternary mercaptopropionic acid (MPA)-capped CuInS_2_ QDs with QYs of 3.3% and studied the influence of various experimental variables, including the precursor concentrations, reaction time, reaction temperature, pH value, and capping ligand used on the luminescent properties of the obtained QDs. These ternary QDs were used to label liver cancer cells. 

Another important aspect to consider is the possible toxicity of this type of QD. Some QDs have been found to be cytotoxic only after oxidative and/or photolytic degradation of their core coatings, demonstrating that core cover greatly improves the biocompatibility of QDs with no observed cytotoxicity even at very high concentration and long-time exposure in cells [21]. Several strategies have been developed to optimize QD stability and biocompatibility. Ternary alloyed QDs represent a promising alternative due to their excellent optoelectronic properties such as: higher QY, tunable emission wavelength controlling Te:Se stoichiometry without significant change in the particle size, wide absorption range, higher chemical and structural stabilities due to decreased interdiffusion, and hardened lattice structure [22]. These advantages may contribute to a decrease in QD cytotoxicity. The stability and cytotoxicity of alloyed QDs have been less studied than binary QDs. Most cytotoxicity approaches and cell internalization studies have been performed on core-shell QDs [23,24,25,26], while the potential cytotoxicity of ternary alloyed QDs remains to be investigated thoroughly [27,28]. Therefore, we begin this work by carrying out a systematic study to obtain water-soluble shell-less alloyed MPA-coated QDs, in a single step, with high QYs and photostability. An important issue is to determine whether the third element in ternary QDs influences their toxicological properties, as well as the influence of surface coating, hydrodynamic diameter, and the particle surface charge.

On the other hand, Förster resonance energy transfer (FRET) mechanism has been widely used in different applications and constitutes the base of a new generation of luminescent sensors. QDs have shown great potential as fluorophores in FRET-based sensing applications, and they have been widely adopted as either energy donors or acceptors [29,30]. Although there are numerous applications of QD-based FRET probes, as far as we know, no studies have been reported demonstrating the use of ternary MPA-capped CdTeSe QDs in FRET systems. 

In this work, we report a systematic study on the synthesis of high QY NIR-emitting MPA-capped CdTeSe QDs without the use of an inert atmosphere and pyrophoric ligands in a single step and in less than an hour. Photostability studies were performed to evaluate the change of fluorescence properties. We used a model based on MPA-capped CdTeSe QDs as energy donors and Cyanine5 NHS-ester (Cy5) dye as an energy acceptor in FRET assays. At pH 7, both species are linked by electrostatic interactions. The effect of QD size on the FRET process between nanoparticles and dye was studied. Finally, cytotoxicity and cell internalization of binary CdTe and ternary CdTeSe QD formulations were tested upon incubation with human cancer cells (HeLa). 

## 2. Materials and Methods

### 2.1. Materials and Characterization

All materials used in the current work were reagents of analytical grade. Cadmium chloride hemi(pentahydrate) (CdCl_2_ + 2.5H_2_O, 81%), potassium tellurite (K_2_TeO_3_, 90+%), 3-mercaptopropionic acid (MPA, 99+%), sodium borohydride (NaBH_4_, 99.99+%), sodium hydroxide (97%), Cyanine5 NHS-ester (Cy5), and fluorescein were purchased from Sigma-Aldrich Chemicals Co. (Toluca, Edo. de Mexico, Mexico). Other reagents (analytical grade) and solvents (spectroscopic grade) were purchased from Sigma-Aldrich Chemicals Co. (Toluca, Edo. de Mexico, Mexico).

The excitation and emission spectra were recorded on a Horiba NanoLog fluorescence spectrophotometer (Kyoto, Japan) using a Xe lamp as the excitation source. QY was determined using fluorescein with a QY of 79% in NaOH 0.1 M as a reference. The absorption spectra were measured using a Varian Cary-100 UV-visible spectrophotometer (Santa Clara, CA, USA). A Jeol JEM2200FS transmission electron microscope (TEM) (Akishima, Japan) was used to examine the appearance and size of nanoparticles. The microscope has spherical aberration correction in a scanning transmission electron microscopy (STEM mode, Akishima, Japan) working at an accelerating voltage of 200 KeV. The elemental composition was determined by energy dispersive spectroscopy (EDS) Oxford (Abingdon, UK), in which the qualitative elemental analysis was made in the STEM mode. Zeta potential was obtained on a Horiba Scientific SZ-100 (Kyoto, Japan) nanoparticle analyzer. All pH measurements were made with a Thermo Scientific pH meter (Waltham, MA, USA). 

### 2.2. Synthesis of MPA-Capped CdTeSe QDs

Water-soluble and highly fluorescent MPA-capped CdTe and CdTeSe nanocrystals were synthesized according to the previously described method with some modifications [31]. Briefly, MPA (0.4 mmol) and CdCl_2_ + 2.5H_2_O (0.4 mmol) were dissolved in 100 mL deionized water in a three-necked flask. Under magnetic stirring, the pH of the mixture was adjusted to 10 by using the drop-wise addition of NaOH solution (1 M). After 5 min of vigorous stirring, 100 mL of a solution prepared from K_2_TeO_3_ and elemental Se of different molar ratios of Te:Se (1:0, 0.75:0.25, and 0.50:0.50) and NaBH_4_ (4.2 mmol) were added into the mixture. After another 5 min of stirring, the flask was attached to a condenser and refluxed at 100 °C. Aliquots were taken from the solution at different refluxing times (30, 45, and 60 min) to obtain QDs of different sizes. The as-prepared MPA-capped QD solutions were concentrated and purified by ethanol precipitation, collected via centrifugation at 3300 rpm, and re-dispersed in water. Concentration of QDs was estimated spectrophotometrically [32]. The QYs of the synthetized MPA-capped QDs were determined through Equation (1):

(1) $$\phi_{x}=\phi_{ST}\left(\frac{Grad_{x}}{Grad_{ST}}\right)\left(\frac{n_{x}^{2}}{n_{ST}^{2}}\right)\;$$

where the subscripts *ST* and *x* denote standard and test, respectively, *ϕ* is the fluorescence quantum yield, *Grad* the gradient from the plot of integrated fluorescence intensity versus absorbance, and *n* the refractive index of the solvent. The standard sample was fluorescein with a QY of 79% in NaOH solution (0.1 M). 

### 2.3. MPA-Capped CdTeSe QDs and Dye Conjugates

Donor MPA-capped CdTeSe QDs were chosen according to the spectral overlap of their emission spectra with the absorption spectra of the acceptor Cy5 dye. The pH of the solution was maintained at 7 using 2-(N-morpholino)ethanesulfonic acid (MES)-buffer (10 mM). At this pH value, the donor and acceptor have negative and positive surface charge, respectively. 

### 2.4. Titration of MPA-CdTeSe QDs with Cy5 Dye

Cyanine5 NHS-ester (Cy5) dye stock solutions were prepared in water (100 µM). 3 mL of QD solution (0.5 µM) were titrated by successive addition of dye stock solution under stirring and their resulting emission spectra were recorded.

### 2.5. CCK-8 Assay

The cytocompatibility of MPA-capped CdTe and CdTeSe QDs was determined through the CCK-8 assay. The CCK-8 is a sensitive colorimetric test that allows a rapid quantification of metabolically active cells in a culture, which consists of the reduction of a WST-8 tetrazolium salt into a water-soluble formazan product (light brown in color). The amount of the colored formazan product is proportional to the number of viable cells [33]. Briefly, HeLa cells with an optical confluence of 80–90% were seeded into 96-well plates (100 µL, 1.5 × 10^4^ cells/well) and grown for 11 h at standard culture conditions (5% CO_2_ at 37 °C) in Dulbecco’s Modified Eagle Medium (DMEM) supplemented with 10% fetal calf serum (FCS), 2 mM l-glutamine, 1% penicillin/streptomycin, 1 mM sodium pyruvate, and 0.1 mM MEM non-essential amino acids (NEAA). Afterwards, the MPA-capped QDs, dissolved in 10 mM phosphate buffered saline (PBS), were pipetted into the cell-containing wells (100 µL, at varying concentrations) and incubated for 8 h. The medium containing the MPA-capped QDs (200 µL) was exchanged with fresh medium (100 µL), and the cells were further incubated to complete 24 h. Then, the culture medium was discarded, and the cells were added to a fresh culture medium (100 µL) containing the CCK-8 reagent (10 µL), followed by gentle shaking for 1 min and incubation for up to 3 h at standard culture conditions. The optical density (OD) of the formazan was measured afterward at 450 nm using an ELISA microplate reader (BIO-RAD model 680, Hercules, CA, USA). The metabolic activity of the cells after exposure to the MPA-capped QDs, represented as the percentage of cell viability, was calculated by normalizing the formazan OD reading from the cells exposed to the MPA-capped QDs regarding control, non-exposed cells (100% viability). The results are the average of six independent experiments. 

### 2.6. Confocal Microscopy

The cellular internalization of the MPA-capped CdTeSe QDs was recorded by using a LEICA TCS-SP5 confocal microscope (LEICA Microsystems Heidelberg GmbH, Wetzlar, Hesse, Germany) equipped with a blue diode (λ = 405 nm) and a pulsed white line laser (WLL, for λ = 488, 561 and 633 nm). The cells were seeded on poly-l-lysine coated glass coverslips (12 × 12 mm) placed inside 6-well plates (3 mL, 5 × 10^4^ cells/well) and grown for 24 h at standard culture conditions. Afterwards, the MPA-capped CdTeSe QDs at the desired concentration were internalized (200 µL, 10^−9^ M), and the incubation protocol was conducted as described for the cytocompatibility experiments. At 24 h of incubation, the MPA-CdTeSe QDs-containing cells were washed and stained for visualization with DAPI (cell nucleus) and Bodipy Phalloidin (cytoplasm). Briefly, 350 mL of paraformaldehyde (4% *w*/*v* in 1× PBS) were added to each coverslip, incubated for 10 min, and washed with 1× PBS. Then, 350 mL of a Triton X-100 solution (0.1% *w*/*v* in 1× PBS) were added, incubated for 10 min, and washed with 1× PBS. Then, 200 µL of Bodipy Phalloidin 650/665 (5% *v*/*v* in 1× PBS) were added, incubated for 20 min in darkness, and washed with 1× PBS. Then, the attached cells were exposed to one drop of ProLong Gold antifade loaded with DAPI and cured for 24 h at −20 °C in darkness. Excitation lines of 405 (emissions at 425–475 nm) and 633 nm (emissions at 650–725 nm) were employed for the visualization of cellular nucleus (stained with 4′,6-diamidino-2-phenylindole (DAPI), from Invitrogen, Carlsbad, CA, USA) and cytoplasm (stained with Bodipy Phalloidin from Invitrogen, Carlsbad, NM, USA), respectively. All experiments were carried out in duplicate.

## 3. Results and Discussion

### 3.1. Synthesis of Near-Infrared MPA-Capped CdTe and CdTeSe QDs

Two important challenges in the synthesis of QDs for their application in biological systems have been to create QDs that (1) are biocompatible and (2) emit in the near infrared. However, many of the synthetic methods reported using organic solvents, which involves a second step for the modification of the QD surface with thiolated ligands to make it water soluble. Although thiol-based ligands have proven stable surface ligands for long-term water solubility, they fail to conserve the high QYs of QDs [34,35]. There is always a significant reduction in the luminescence efficiency following the transition from organic solvents into water (QY is usually below 20%) [36]. For this reason, the direct aqueous synthesis of MPA-capped QDs using a single-step and ambient atmosphere methodology was performed. The synthesis conditions were modified with the objective to optimize the QY of the QDs. Then, QDs of CdTeSe were obtained at different molar ratios of Te:Se (1:0, 0.75:0.25, and 0.50:0.50) and different reaction times (15, 30, 60, and 90 min). Figure S1 shows images of the as-prepared QDs under ambient light and UV light. A clear red-shift in the emission wavelength is seen for longer reaction times, indicating that larger QDs have formed.

#### Characterization by UV-VIS and Fluorescence Spectroscopy

**Figure 1:**
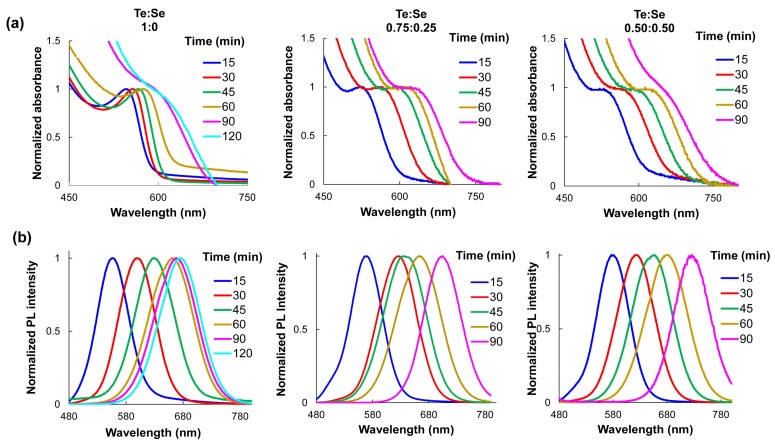
Evolution of (**a**) absorption and (**b**) fluorescence spectra of mercaptopropionic acid (MPA)-capped CdTeSe quantum dots (QDs) in water at pH 8 obtained at different molar ratios of Te:Se (1:0, 0.75:0.25, 0.50:0.50) and different time reactions.

Absorption and fluorescence spectra of the MPA-capped QDs re-dispersed in water at pH 8 were obtained. As can be appreciated in Figure 1, when the reaction time increases and the Te proportion is lower, the emission wavelengths shift to the near-infrared zone. Unlike binary QDs, the growth kinetics of alloyed CdTeSe QDs are dependent upon three chemical reactants (Cd, Te, and Se) and their relative concentrations in the reaction [12]. It has been proven that elemental Te is considerably more reactive than Se toward Cd under rapid nucleation and that equilibrium between Te and Se are reached at higher reaction times [10]. The change in the Te and Se composition during QD growth was reflected on the QDs emission wavelengths.

The mechanism of the QDs’ formation can be described as follows; during the first step MPA coordinates with Cd^2+^ ions to form the Cd-MPA complex and to prevent the deposition of non-soluble Cd compounds. The addition of NaBH_4_ results in the reduction of TeO_3_^2−^ to Te^2−^ and the reduction of elemental Se to Se^2−^. The precursor of Te (Te^2−^) rapidly reacts (twice as fast as Se^2−^) resulting CdTe-rich nuclei QDs initially. As the reaction progresses, CdSe is formed on the core, and an alloyed structure is produced. MPA ligands tightly bind to the QDs’ surfaces.

**Figure 2:**
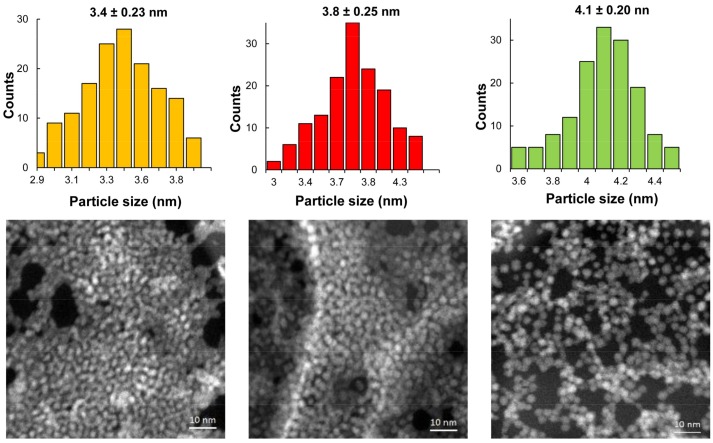
Scanning transmission electron microscopy (STEM) images obtained by Z contrast of the MPA-capped QDs at different molar ratios of Te:Se (1:0, 0.75:0.25, 0.50:0.50) and time reaction of 60 min. Above: the corresponding histogram of size distribution.

Similar to the change in the emission wavelength, QYs become larger as the reaction time increases from 15 to 60 min and the Te:Se molar ratio decreases (Table S1). The highest QY, 84%, was obtained with a molar relation of 0.75:0.25 of Se:Te and a reaction time of 60 min. These are remarkable results since few investigations have obtained comparable QY values for water-soluble QDs [37]. The MPA-capped QDs’ diameters were estimated using the absorption peaks and the empirical equation previously reported by Peng [32] at different reaction times (see Table S1). STEM images of the prepared MPA-capped CdTe and CdTeSe QDs (Figure 2) indicate uniformly dispersed, spherical QDs. The results obtained from the analysis of the size distribution histograms of the QDs show that the average crystal size was 3.4 ± 0.23, 3.8 ± 0.25, and 4.1 ± 0.2 nm, confirming the results obtained by the Peng regression. 

Nanoparticle composition was further examined by EDS analysis. The presence of Cd, Te, and Se was confirmed by the presence of characteristic peaks of these elements (Figure S2). The remaining signals correspond to lattice elements of the sample support.

**Figure 3:**
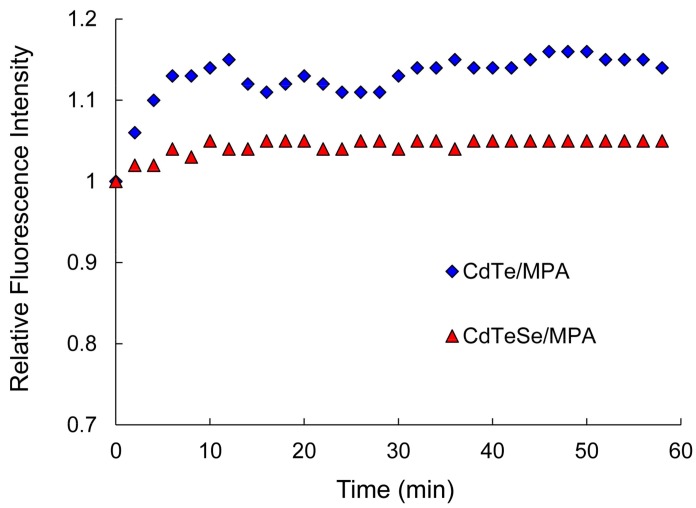
Photostability of the MPA-capped CdTe and the MPA-capped CdTeSe QDs in water at pH 8 under UV light irradiation.

Moreover, the obtained QDs dispersed in the phosphate buffer (pH 8) showed high optical stability when irradiated under xenon ion laser at 365 nm UV light for 60 min. Figure 3 shows the dependence of the relative fluorescence intensity of the MPA-capped CdTe and MPA-capped CdTeSe QDs with the UV irradiation time. Previous studies have shown that unsaturated Te and Se atoms on QDs’ surfaces may be oxidized under UV light exposure, which may lead to possible quenching of QDs’ fluorescence [38]. The as-prepared QDs (binary and ternary) showed no decrease in fluorescence intensity. In fact, a photo enhancement of 15% was observed for the MPA-capped CdTe QDs in the first 10 minutes, while the fluorescence of the alloyed MPA-capped CdTeSe remained practically constant throughout the irradiation time interval. The differences in luminescence can therefore be attributed to different surface properties [6]. The alloyed MPA-capped CdTeSe QDs with gradient distribution of components consisting of Te-rich inner cores and Se-rich outer shells contain fewer trap sites or quenching defects on the surface.

The photo brightening of the MPA-capped QDs after UV irradiation has been associated with the photo-adsorption of water molecules onto nanocrystals’ surfaces providing restoring functions on the QDs’ surfaces, eliminating photoluminescence (PL)-quenching defects or trap sites [39]. These results confirm that there are fewer trap sites or quenching defects on the surfaces of ternary QDs. Based on this behavior, the MPA-capped CdTeSe QDs were selected for further FRET studies.

### 3.2. Förster Resonance Energy Transfer FRET Studies

**Figure 4:**
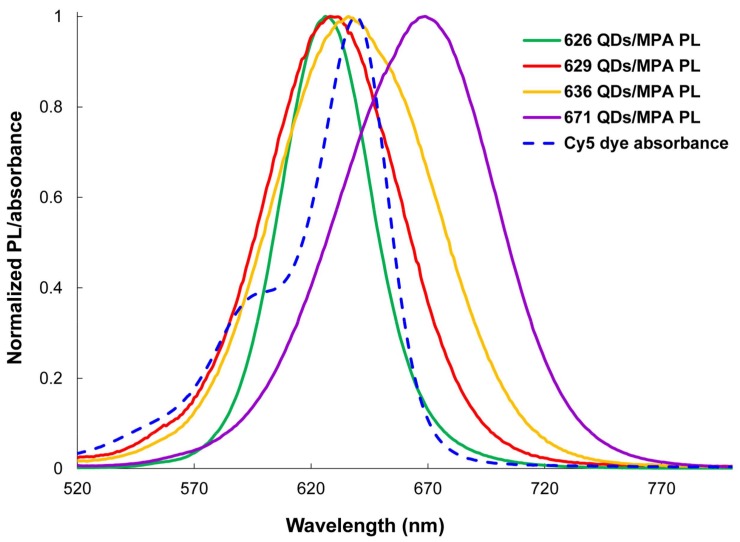
Spectral overlap of the donor MPA-capped CdTeSe QDs emissions and the acceptor Cy5 dye absorption. The solid lines represent the emission spectra of the donors (λ_em_ = 626, 629, 636, and 671 nm) and the dotted line represents the absorption spectrum of the Cy5 dye (λ_em_ = 639 nm).

The potential of QDs as fluorescent energy donors in FRET assays was explored by designing four systems of MPA-capped CdTeSe QDs (QDs626, QDs629, QDs636, and QDs663) as energy donors and Cy5 dye as energy acceptors. According to the Förster resonant energy transfer theory, a condition that affects the efficiency of energy transfer is the overlap between the donor emission spectrum and the acceptor absorption spectrum. The absorption spectrum of the Cy5 dye and emission spectra of the four MPA-capped CdTeSe QDs (spectra are normalized) are shown in Figure 4. As can be seen, the emission spectra of the MPA-capped CdTeSe QDs and the absorption spectrum of the Cy5 showed a large spectral overlap.

### 3.3. Selection of the Optimal Conditions for the FRET Process

**Figure 5:**
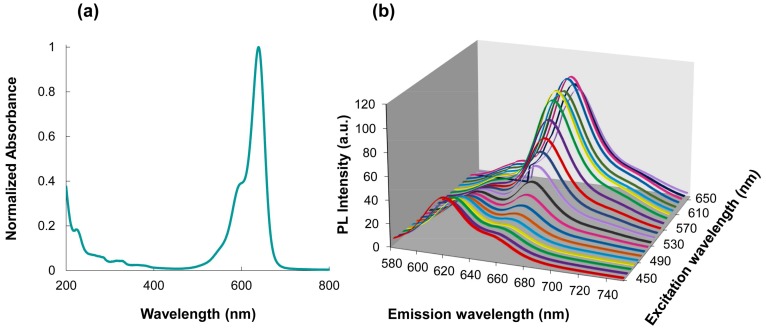
(**a**) Absorption spectrum of the Cy5 dye and (**b**) the MPA-capped CdTeSe-Cy5 conjugate 3D spectra.

The optimal wavelength to excite the FRET pair is the one for which only the donor is excited, and the acceptor excitation is minimal. As can be observed in the absorption spectrum of the dye in the Figure 5a, this wavelength is 450 nm. In Figure 5b, a three dimensional (3D) spectrum produced for the MPA-capped CdTeSe and the dye solution with a molar ratio of 1:20, respectively, is shown. The 3D spectra in Figure 5b show that the optimum excitation wavelength of the QDs is at 450 nm, while the optimum excitation wavelength of the dye is 630 nm.

### 3.4. Zeta Potential

**Figure 6:**
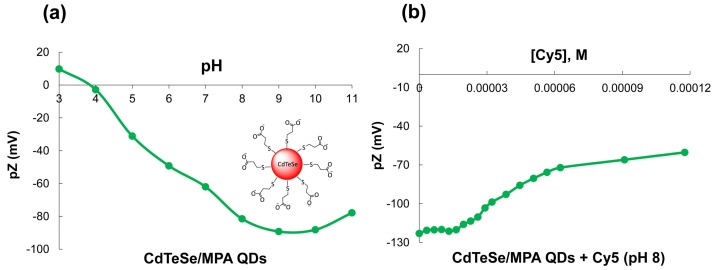
(**a**) Potential Z of the MPA-CdTeSe QDs at different pH values and (**b**) the titration of the MPA-CdTeSe QDs with the Cy5 at pH 8.

With the objective to corroborate the surface charge on the QDs and the electrostatic interactions between the MPA-capped CdTeSe QDs and the Cy5 dye, Z potential was determined by varying the pH of the QDs (Figure 6a) and adding successive amounts of the Cy5 dye (Figure 6b).

The MPA-capped QDs have negative charge between pH 4–11 (Figure 6a), due to carboxylate groups on their surfaces. An isoelectric point is localized at pH 4 coinciding with the pKa of the carboxylic acid. The successive additions of the dye to the MPA-QDs, lead to a decrease in the QDs’ negative surface charge (−60 mv) upon addition of increasing amounts of the Cy5 dye, indicating complex formation (Figure 6b). This behavior confirms the electrostatic interactions between the positively-charged dye and the negatively-charged nanoparticles, decreasing the total charge of the MPA-QDs. The MPA-capped CdTeSe QDs bond with the Cy5 dye via electrostatic interactions between their carboxylate groups and the iminium group of Cy5 dye. In the phosphate buffer (pH 8), stable conjugates are formed.

### 3.5. MPA-Capped CdTeSe QDs’ Titrations of Different Sizes with Cy5 Dye

**Figure 7:**
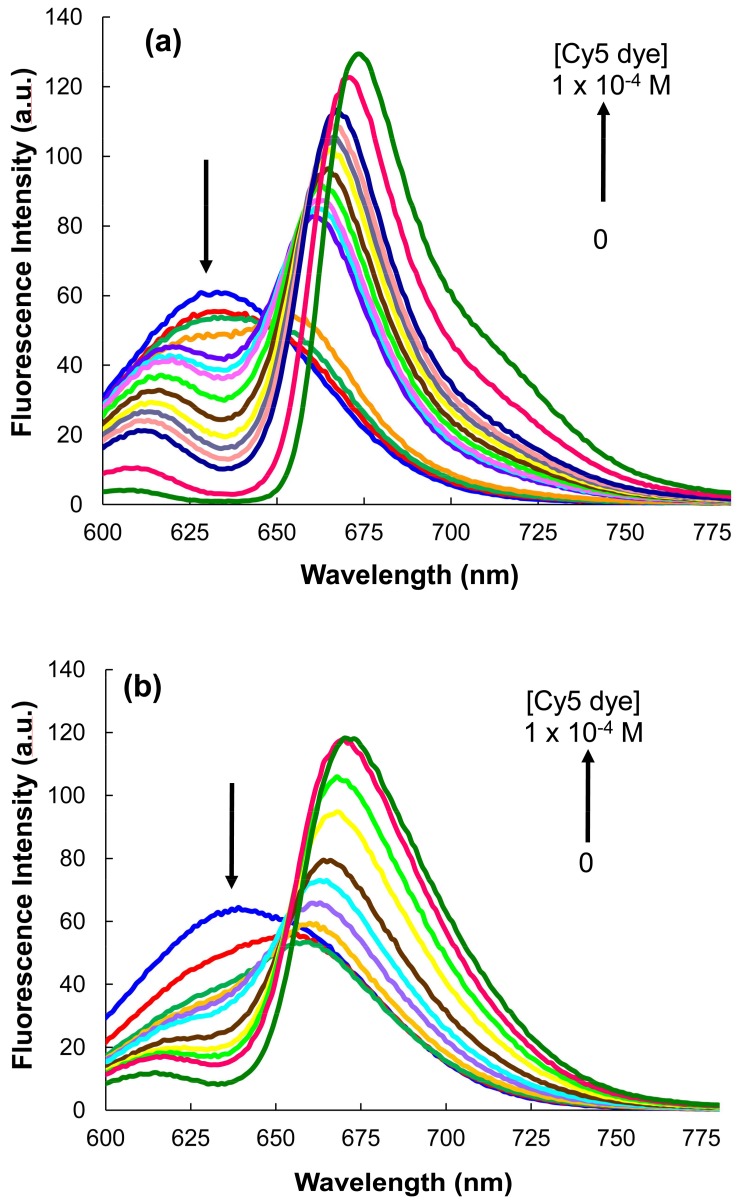
Titration emission spectra of the MPA-capped CdTeSe QDs with the Cy5 dye. (**a**) QDs629 and (**b**) QDs636.

The influence of the QDs’ sizes on the FRET process was studied by successive titrations of different sizes of QDs (QDs626, QDs629, QDs636, and QDs671) with the Cy5 dye. A decrease in the QDs’ emissions and the enhancement of the Cy5 dye emission was only seen for QDs629 and QDs636, indicating a local interaction between the QDs and the Cy5 dye, as well as a FRET process for these conjugates. For the conjugates between QDs626 and QDs663, = the typical behavior of a FRET process was not shown. Figure 7 shows the emission spectra of the successive titration of the MPA-capped CdTeSe QDs (629 and 636) with the Cy5 dye (λ_ex_ = 450 nm).

Figure S3 illustrates the PL signals for the two sets of 629- and 636-nm-emitting QDs, as a function of the Cy5/QD molar ratio. The plots agree well with the experimental observations for the progressive donor PL quenching compared to the acceptor PL enhancement with increasing amounts of the Cy5 dye. Graphic analysis also shows that the enhancement of the fluorescence intensity of the Cy5 dye begins to show signs of saturation at a molar ratio of 50:1. The results coincide with previous studies, which have reported saturation molar ratios larger than 10:1 to 50:1 [40,41]. 

The energy transfer process between the QDs and the Cy5 dye was confirmed for the QD629-Cy5 and QD636-Cy5 FRET pairs. The fact that *R*_0_ (the Förster distance) and *r* (the distance between the QDs and the dye) values are in the range of 2–8 nm and that the required condition, 0.5*R*_0_ < *r* <1.5*R*_0_, was fulfilled, indicate the existence of the energy transfer from the QDs to the Cy5 dye in these cases [42]. For the QD626-dye and QD671-dye FRET pairs, the condition is not fulfilled. The MPA-capped QD629-Cy5 and MPA-capped QD636-Cy5 donor-acceptor pairs had good results with high efficiency, overlap area, and binding constant (Table 1). Based on these results, the QD*s*629 and QD*s*636 can be used in FRET assays with the Cy5 dye in biological applications.

### 3.6. Citocompatibility of the CdTeSe/MPA QDs

**Figure 8:**
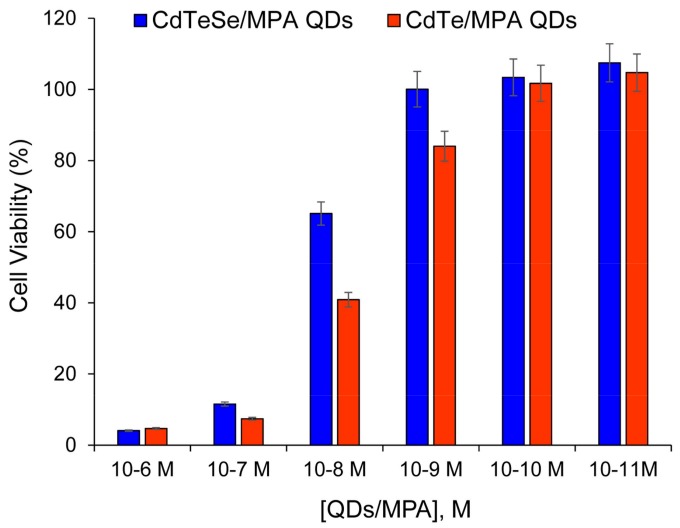
Viability of human cancer cells (HeLa) cells after exposure to the MPA-capped CdTe and CdTeSe QDs.

Figure 8 shows the viability of HeLa cells after exposure to the MPA-capped CdTe and MPA-capped CdTeSe QDs as a function of particle concentration. This figure reflects that both QDs display a dose dependent cytocompatibility typical of nanomaterials, depicting a LC50 (lethal concentration for 50% of the cells) of about 10^−8^ M and a MinTC (minimum toxic concentration) of ca. 10^−9^ M, below which cell growth inhibition appears to be negligible. These results are in good agreement with those presented by Hu et al. [43], who did not observe cytotoxicity for four CdSe/ZnS QDs coated with PEG within the concentration range of 10–100 nM upon internalization to human hepatocellular carcinoma (HepG2) cells [42]. The authors attributed this outstanding outcome to the structure of the ZnS shell and the PEG coating, which are expected to avoid the release of Cd from the QDs’ cores. In our case, such a biocompatible coating is rendered by the MPA, which also endowed the QDs with a zeta-potential of about −65 mV (see Figure 6), protecting the cells from the membrane disruption phenomenon commonly observed upon their interaction with positively-charged compounds. In our results, better cytocompatibility is found for the ternary QDs than for the binary QDs, which is in agreement with the higher stability of the ternary QDs (Figure 3).

Assessing the QDs’ potential toxicity is not a simple matter. A number of publications agree that the main toxicity sources in Cd^2+^-containing QDs are Cd^2+^ ion release and reactive oxygen species (ROS) production. Kauffer et al. [44] compared the ability of ternary alloyed QDs (mercaptosuccinic acid (MSA)-capped CdSeS QDs) versus binary QDs (MSA-CdSe) to generate light-induced ROS. The binary QDs produced ·OH radicals immediately, whereas the ternary QDs required extensive irradiation times and presented less photobleaching. The third element in alloyed QDs plays a major role in providing a higher structural and chemical stability; this may result not only in less ROS production but also in a decrease of Cd^2+^ release.

### 3.7. Cell Internalization of the MPA-Capped CdTeSe QDs

**Figure 9:**
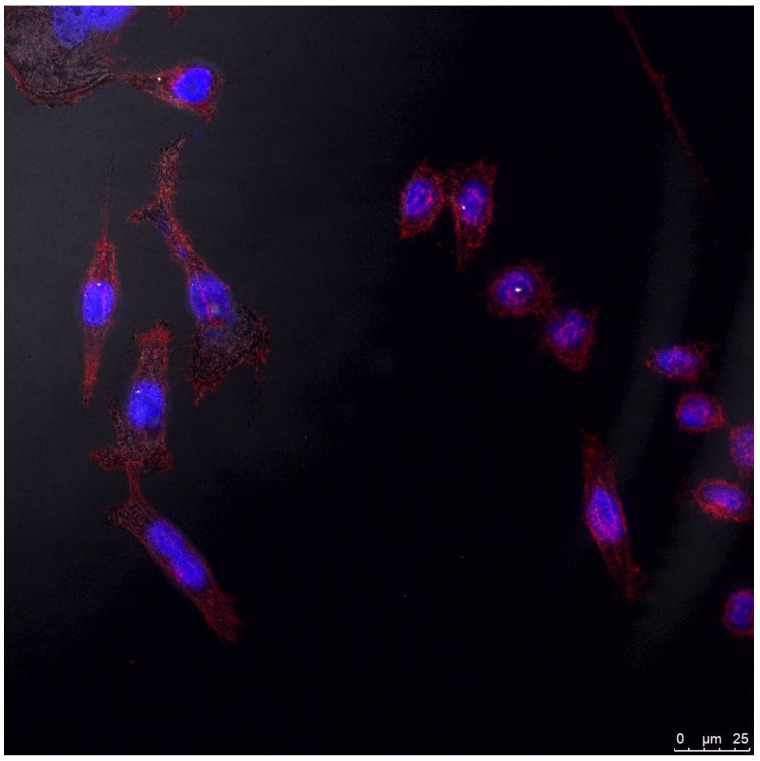
Confocal microscopy image in reflection mode of HeLa cells upon exposure to the MPA-capped CdTeSe QDs. Stained in blue and red are the cell nucleus and cytoplasm, respectively. QDs are observed in reflection mode as black dots all over the cytoplasm.

Figure 9 shows the internalization of the MPA-capped CdTeSe QDs at 6 h post-internalization at a concentration of 10^−9^ M. This figure reflects two features that are worthy of discussion. Firstly, the displayed image shows a high population of cells presenting a healthy appearance in presence of the QDs after internalization, even during mitosis when they are known to be especially sensitive to any external stimuli [45]. This finding confirms the CCK-8 results identifying the MPA-capped CdTeSe QDs as cytocompatible. Second, the QDs homogeneously accumulated all over the cytoplasm (depicted as black dots in reflection mode), demonstrating these ternary materials as suitable and cytocompatible candidates for cell staining.

## 4. Conclusions

We have synthesized high quantum yield and stable NIR-emitting alloyed MPA-capped CdTeSe QDs that are water soluble using a facile and direct modified synthetic route. PL properties of the QDs were influenced by reaction time and Te:Se composition. The MPA-capped CdTeSe QDs exhibited a red-shifted emission, higher QYs, and better stability and cytocompatibility than corresponding MPA-capped CdTe QDs. By careful selection, different sized MPA-capped CdTeSe QDs (energy donors) and Cy5 dye (energy acceptor) were used for FRET assays. All factors for effective FRET were studied, such as QD size, proximity between the donor and the acceptor pair (electrostatic interactions), and high spectral overlap. The occurrence of FRET was confirmed in the conjugates of MPA-capped CdTeSe 629 nm QDs with Cy5 and in MPA-capped CdTeSe 636 nm QDs with Cy5 with good efficiency. 

Very importantly, as depicted by the CCK-8 assay and confocal microscopy, the QDs were demonstrated to be highly cytocompatible and capable of ordering homogeneously and in high proportions all over the cytoplasm. The outstanding cytocompatibility of the QDs studied, the good efficiency of the FRET process, and their stability make the proposed system a good candidate for the development of FRET-based analytical tools such as fluoroimmunoassays.

## Figures and Tables

**Table 1:** Calculated overlap integrals (*J*), FRET efficiencies (*E*), Förster distances (*R*_0_), distance between the donor and the acceptor (*r*), and binding constant (*K*) obtained by Equations (S1)–(S4) (Supplementary Materials).

MPA-QDs	λ_em_ (nm)	D_UV_ (nm)	*J* (cm^3^Lmol^−1^)	*E*	*R*_0_ (nm)	*r* (nm)	*K*
QD629	629	3.48	2.01 × 10^−12^	0.96	4.37	2.58	2.30 × 10^4^
QD636	636	3.63	1.88 × 10^−12^	0.88	4.41	3.14	8.24 × 10^4^

## Supplementary Materials

The following are available online at http://www.mdpi.com/2079-4991/8/4/231/s1. Figure S1: Images of the QDs under ambient light and UV light. Figure S2: EDS spectra of the CdTeSe QDs at different molar ratios of Te:Se (1:0, 0.75:0.25, 0.50:0.50) and time reaction of 60 min. Figure S3: Experimental values for the donor relative PL intensity decay (red line) and acceptor PL intensity gain (blue line) versus molar ratio [Cy5]/[QD]. Table S1: Optical characterization of the CdTeSe QDs. FRET calculations and Equations (S1)–(S4).
