# Supplementary material for: NIR-Emitting Alloyed CdTeSe QDs and Organic Dye Assemblies: A Nontoxic, Stable, and Efficient FRET System
Source: Nanomaterials (Basel). 2018 Apr 11;8(4):231. doi: 10.3390/nano8040231 (PMC5923561; doi:10.3390/nano8040231)
Supplement: Supplementary file 1 [file nanomaterials-08-00231-s001.pdf]

Supporting Information

NIR-Emitting Alloyed CdTeSe QDs and Organic Dye Assemblies: A Nontoxic, Stable, and Efficient FRET System

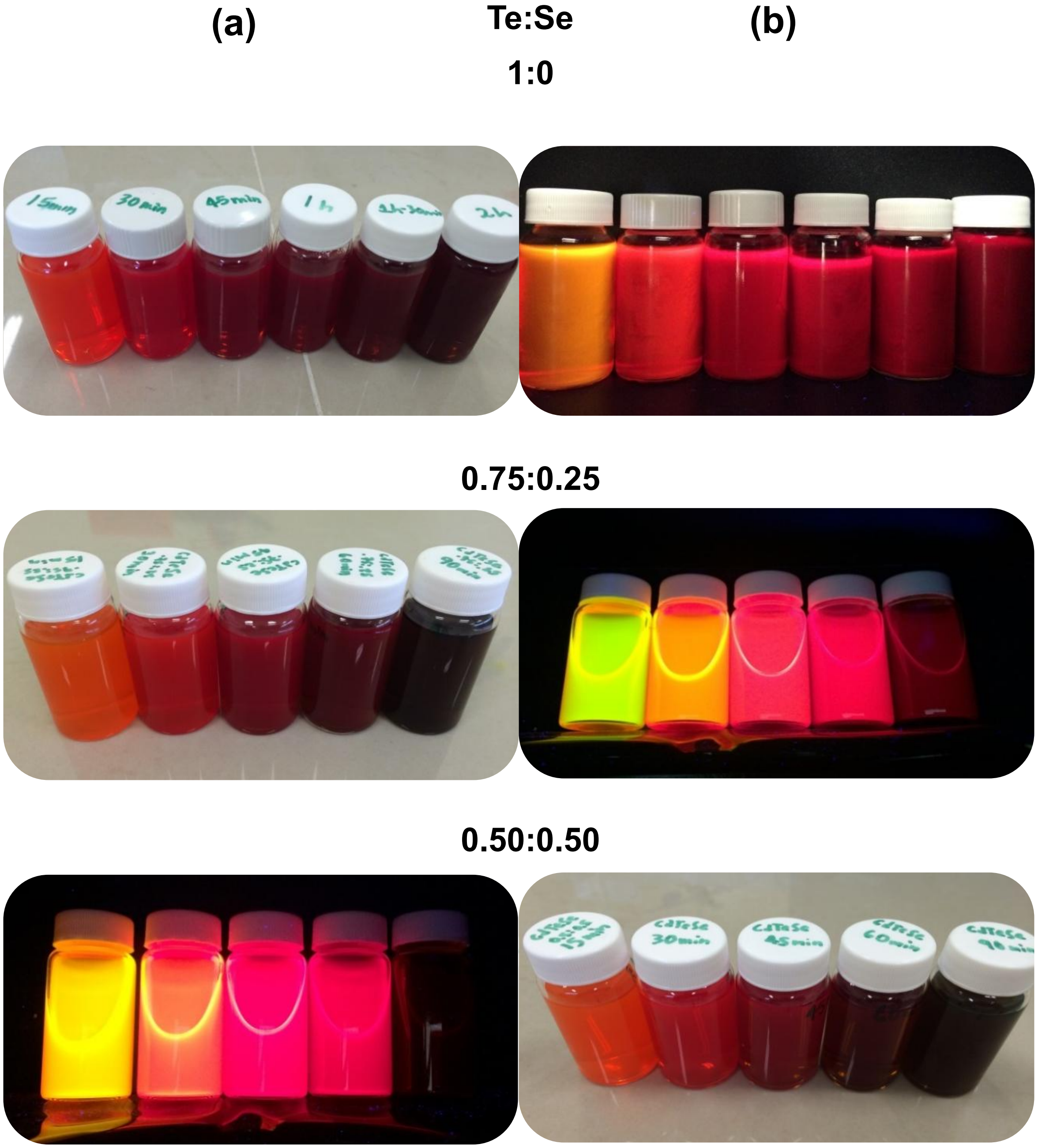

Figure S1. Images of QDs under ambient light and UV light.

Table S1. Optical characterization of CdTeSe QDs.

Molar Ratio Te:Se	Time reaction (min)	d (nm)*	λ_{em} (nm)	QY
**1:0**	15	2.42	554	26
	30	3.09	594	49
	45	3.58	628	55
	60	3.59	663	59
	90	3.61	668	53
	120	3.66	674	55
**0.75:0.25**	15	2.85	569	21
	30	3.38	626	51
	45	3.63	636	73
	60	3.82	671	84
	90	3.96	704	46
**0.50:0.50**	15	2.96	580	31
	30	3.48	629	53
	45	3.67	657	73
	60	3.96	680	80
	90	4.28	724	11

* Diameter was estimated using the absorption peaks and the empirical equation previously reported by Peng [1].

[1] Yu, W.W.; Qu, L.H.; Guo, W.Z.; Peng, X.G. Experimental determination of the extinction coefficient of CdTe, CdSe and CdS nanocrystals. *Chem. Mater.* **2003,** *15*, 2854-2860.

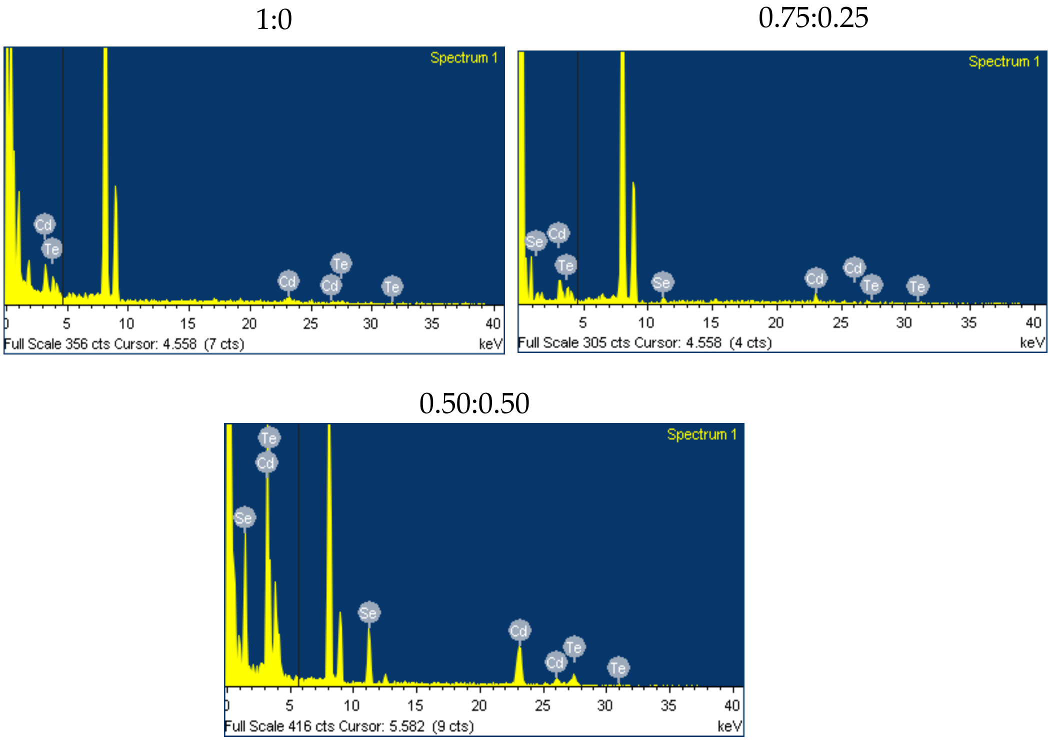

Figure S2. EDS spectra of CdTeSe QDs at different molar ratio Te:Se (1:0, 0.75:0.25, 0.50:0.50) and time reaction of 60 min.

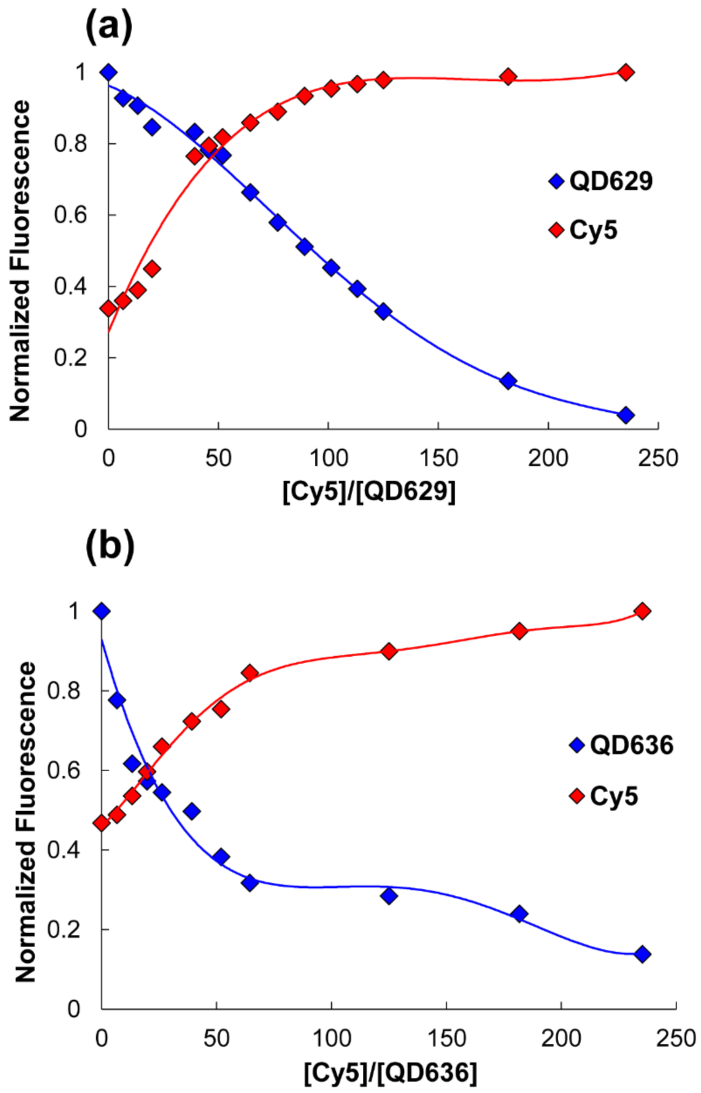

Figure S3. Experimental values for the donor relative PL intensity decay (red line) and acceptor PL intensity gain (blue line) versus molar ratio [Cy5]/[QD].

FRET calculations

FRET efficiency (E):

$$E = 1 - \frac{F_{DA}}{F_D} = \frac{R_0^6}{R_0^6 + r^6} \qquad (S1)$$

where F_{DA} is the integrated donor fluorescence in the presence of the acceptor and F_D is the integrated donor fluorescence alone, R_0 is the Förster distance, which is the

separation distance that yields 50 % energy transfer efficiency and r is the distance between donor and acceptor.

Förster distance (R_0):

$$R_0^6 = 8.79x10^{-25}K^2n^{-4}\phi J(\lambda) \qquad (S2)$$

where K^2 is the dipole orientation factor (for random orientation; 2/3), n is the refractive index of the medium, φ is the fluorescence quantum yield of the donor in the absence of acceptor and $J(\lambda)$ is the integral of the spectral overlap.

Overlap Integral $J(\lambda)$:

$$J(\lambda) = \int_0^\infty F_D\,(\lambda)\varepsilon_A(\lambda)\lambda^4 d\lambda \qquad (S3)$$

The overlap integral is a quantitative measure of the donor-acceptor spectral overlap over all wavelengths λ; $F_D(\lambda)$ is the fluorescence intensity of the fluorescence donor at given wavelength, and $\varepsilon_A\,(\lambda)$ is the molar absorptivity of the acceptor at given wavelength.

Binding constant (K):

Stern-Volmer modified (static quenching) equation,

$$\frac{1}{F_0 - F} = \frac{1}{F_0} + \frac{1}{KF_0[Q]} \qquad (S4)$$

where F_0, is the fluorescence intensity in the absence of the quencher and F is the fluorescence intensity in presence of quencher, [Q] is quencher concentration and K is the binding constant of donor-acceptor conjugate.
